# Perception of Policy and Environmental Action to Promote Healthy Behaviors in African American Communities

**DOI:** 10.3390/ijerph14030271

**Published:** 2017-03-07

**Authors:** Clifton Addison, Brenda W. Campbell Jenkins, Monique White, Frances Henderson, Dorothy J. McGill, Donna Antoine-LaVigne, Marinelle Payton

**Affiliations:** 1 Center of Excellence in Minority Health and Health Disparities, School of Public Health, Jackson State University, 350 West Woodrow Wilson Drive, Suite, 2280, Jackson, MS 39213, USA; brenda.w.campbell@jsums.edu (B.W.C.J.); monique.s.white@jsums.edu (M.W.); henfra@att.net (F.H.); Djceo@ibshealthy.com (D.J.M.); donna.antoine-lavigne@jsums.edu (D.A.-L.); marinelle.payton@jsums.edu (M.P.); 2 Jackson Heart Study Community Outreach Center, School of Public Health, Jackson State University, 350 West Woodrow Wilson Drive, Suite, 2900B, Jackson, MS 39213, USA; 3 Jackson Heart Study Graduate Training and Education Center, School of Public Health, Jackson State University, 350 West Woodrow Wilson Drive, Suite, 2900B, Jackson, MS 39213, USA

**Keywords:** public health, disparities, policy, cardiovascular disease, African Americans

## Abstract

The present study aimed to examine the perceptions of African American communities regarding the involvement of political leaders in facilitating policy and environmental change promoting healthy eating and physical activity. We selected the Metro Jackson Area comprised of Hinds, Madison and Rankin Counties because it is a combination of urban and rural communities. The sample consisted of 70 participants from seven sites. A total of seven focus groups were asked to respond to one question to assess political leaders’ involvement in healthy living: “When you think about your political leaders that you have in the Jackson, Mississippi area, do any of them promote healthy eating and physical activity?” Focus groups consisted of six to 12 participants and were asked to comment on their participation in physical activity. The focus group interviews were digitally recorded. The recorded interviews were transcribed by a professional transcriptionist. Community members could not recollect much participation from political leaders in the health prevention/intervention efforts. In each of the counties, there was evidence that there was some involvement by local politicians in health promotion issues, but not on a large scale. In conclusion, making healthy foods and products available in neighborhood stores has long been associated with healthy behaviors and positive health outcomes. This can make a difference in the Mississippi communities where supermarkets are not accessible and health disparities abound.

## 1. Introduction

As Mississippi struggles to improve cardiovascular health status and eliminate health disparities, particularly among African Americans, it is imperative that politicians become engaged in the drive to effect new policies at the local, state, and federal levels; new policies are needed to facilitate changes in lifestyle and practices that would be important components of a strategic healthy lifestyle plan. Obesity is a major risk factor that affects the health of many communities in the nation. The Centers for Disease Control and Prevention (CDC) reported that more than two-thirds of American adults and one-third of American youth are obese or overweight, and obesity is a major contributor to much of the morbidity and mortality in the U.S. [1]. For some communities, the rise in obesity is associated with two main factors: lack of access to healthy foods and inadequate physical activity. Political leaders can influence changes in health statistics by promoting the enactment of laws and policies to influence environmental and cultural changes that can tackle the obesity epidemic and support healthy living.

There have been some recent policy changes by governmental agencies that have been designed to address the health impact of risk factors. In 2011, the U.S. Department of Agriculture proposed nutritional standards for the national school breakfast and lunch programs to ensure that they were meeting the nutritional guidelines recommended by scientists to promote healthy eating [2]. In 2010, the federal Affordable Care Act (ACA) was passed, and this law required that retail food establishments and vending machine operators should reveal details about calorie and nutritional contents of foods that they are making available to the public. Many of the federal agricultural policies have been developed to change negative eating behaviors into more positive ones at the local and national levels. Local and state governments can use their influence to promote healthy living policies, by initiating environmental changes through new zoning or licensing laws and other appealing programs that can influence additional neighborhood changes, setting the stage for improved dietary practices and increased involvement in physical activity [3].

Policy changes at the level of the Office of the President of the United States represent an example of changes at the highest level that can be implemented to influence positive healthy behaviors. As First Lady, Michelle Obama made a commitment to help to reduce childhood obesity. She committed herself to helping children grow into adulthood at a healthy weight when she initiated the “Let’s Move” national campaign. The “Let’s Move” campaign was designed to bring together public and private resources that can be used to provide schools, families, and communities with the means necessary to motivate children to modify negative dietary practices and inadequate physical in seeking a better and healthier lifestyle [4].

Local politicians do not generally hold the promotion of healthy environments as a top priority. The *Wall Street Journal* conducted a research study where experts were asked to provide their perception on the role that “the government should play in reducing obesity. The journal believed that the government should become concerned enough to develop new procedures to treat obesity” [5]. Politicians should assume a role in the promotion of a healthy lifestyle because they hold an important platform that can be used to promote and direct changes, in addition to having the status to solicit funding that can facilitate changes in food environments and provide increased opportunities for physical activity [6]. A recent study showed that there was strong support among Mississippi residents for a diverse set of policies that could impact healthy eating and physical activity behaviors. This would especially be beneficial for those communities located in counties with the highest levels of obesity. The study showed that there is abundant support for the implementation of policies that would promote healthy eating and physical activity [7].

According to the Public Health Law Center [3], politicians can promote policies that can be used to promote healthy eating environments and assure easy access to healthy foods. It is also feasible that policy can be implemented to influence worksite wellness. This would be a direct strategy that can lay a strong foundation for inspiring healthier living. The objective of this study was to examine the perceptions of African American communities regarding the involvement of political leaders in facilitating policy and environmental change promoting healthy eating and physical activity.

## 2. Methods

### 2.1. Focus Group Participants and Selection Criteria

Data for this analysis were collected from 70 residents at seven sites in the Metro Jackson, Mississippi Area (Hinds, Madison, and Rankin County, a combination of urban and rural communities) between May 2014 and February 2015. Seven focus groups were asked to respond to one question that assessed their political leaders’ promotion of healthy living (see Table 1). Each Focus group consisted of six to 12 participants who were also asked to respond to six demographic questions that provided a description of the characteristics of the participants. The sample of 70 participants represented the following six organizations: Innovative Behavioral Services (10), Ntense Fitness (8), Sweet Rest Baptist Church (13), Progressive Baptist Church (two groups totaling 21), Canton Multi-Purpose Building (12) and Asbury United Methodist Church (6). These counties were selected due to their high rates of residents who were physically inactive [8]. Poor dietary practices and lack of physical activity increase the risk for diseases such as diabetes, cardiovascular disease and obesity [9]. Hinds County is the largest county in the state. It is the location of the state capitol. Madison and Rankin Counties are rapidly growing rural and suburban counties [10]. Participants were recruited by networking with community partners, local churches, fitness centers and local government entities as well as dissemination of flyers and announcements at churches, and town hall events within each of the counties. Additional details about the composition of the focus groups are chronicled elsewhere.

### 2.2. Focus Group Procedures

To elicit community members’ knowledge regarding genetics, behavioral, biological and environmental factors relating to obesity, cardiovascular disease, and mortality, the program administrators developed a Community Questionnaire designed to collect demographic data such as: county of residence; gender; age; income; education; and employment. The questionnaire data collection focused on data regarding the community residents’ perceptions of political leaders’ promotion of healthy living.

This study focused on the community members’ perceptions about political leaders’ promotion of healthy living. A qualitative study design was selected because it was best suited to enable our researchers to strive for an understanding of the whole [11] by engaging the community members to explore their own needs and aspirations [12] in a manner that acknowledges the community as a unit of identity, a major principle of community-based participatory research (CBPR) [13]. Establishing the value of African American life can more effectively initiate discussions to address issues that improve the quality or quantity of that life [14]. These focus group interviews allowed us to hear multiple voices at one sitting [15], and participants got an opportunity to discuss issues and question and build upon one another’s answers [16].

### 2.3. Ethics Statement

All community members gave their informed consent for inclusion before they participated in the study. The study was conducted in accordance with the Institutional Review Board at Jackson State University, Jackson, Mississippi, and the protocol was approved by the IRB (2014-2015). All participants were asked to complete and sign a consent form, thus abiding by the informed consent process. Participants were reminded that their participation was voluntary and provided instructions for protecting confidentiality. The focus group interviews were digitally recorded. The recorded interviews were transcribed by a professional transcriptionist.

### 2.4. Data Analysis

We analyzed the focus group data using Interpretive Phenomenology in order to arrive at interpretive descriptions of common practices and shared meanings that could reveal, enhance or extend our understanding of how participants perceived specific health practices and possibilities. We adapted Diekelmann’s seven stage process of data analysis [11]. Audio-recordings were transcribed verbatim into Microsoft Word (Microsoft, Albuquerque, NM, USA. A detailed description of the coding process and data management is available elsewhere. Similarities and differences in political leaders promotion of healthy living were identified within individual interview sites in the three counties (Asbury, Canton, Innovative Behavioral Services (IBS), Ntense, Progressive I, Progressive II and Sweet Rest), and then similarities and differences were examined between counties (Hinds, Madison, and Rankin Counties). A data file worksheet, by setting, for each of the three counties (Hinds, Madison and Rankin) was developed. Writing group members read through each county-specific focus group data file, and listed, on the worksheet, their interpretations for use in developing key themes for each focus group, according to the response to the questions. For this study, the data files were separated for interpretation according to the question addressed in the focus group sessions about political leaders promoting healthy eating and physical activity. Writing group members reached consensus on their interpretations of the responses, and/or reconciled differences via discussion.

## 3. Results

### 3.1. Focus Group Demographics

A total of 70 focus group members participated in interviews about dietary practices and possibilities for healthy eating. They were from the three counties that encompass the Jackson Heart Study: Hinds (*n* = 28); Madison (*n* = 27) and Rankin (*n* = 13). We did not have county data for two participants. Of the 70 participants, 56 were female, 14 were male; we are missing gender data for one participant. Participants’ ages ranged 18–60 and over; most participants (*n* = 30) were age 60 and over, two-thirds (66%) were 50 years of age and older. In terms of income, we are missing data for eight participants; of the remaining 62, 20% earned less than $21,000 per year. Income for slightly more than half of the participants ranged from $21,000 to $39,000 per year. Participants with incomes of $40,000 to over $60,000 per year totaled 16 (26.0%).

For most participants (78%), their educational level ranged from some college to a Master’s degree or higher, leaving 21% with a high school diploma, and one for whom we are missing data. While 30 of the 70 focus group participants were retired, 34 (49%) were employed, less than 5% were unemployed, and two were students. In addition, we are missing employment data for one participant.

### 3.2. Focus Group Responses

Themes and sub-themes of the focus group responses are reported in Table 2 according to the question about political leaders’ promotion of healthy eating and physical activity reported in Table 1. The responses to this question help us to begin to explore this tri-county African American community’s perception of political leaders’ promotion of healthy eating and physical activity in local communities. Community members from Hinds, Madison, and Rankin Counties could not recollect much participation from political leaders in the health prevention/intervention efforts. In each of the counties, there was evidence that there was some involvement by local politicians in health promotion issues, but not on a large scale.

For Hinds County, there were several “no” responses, as well as examples of political leader’s promotion of physical activity: “Council was instrumental in getting a walking trail in the neighborhood. Our new mayor has participated in various health walks through the community”. The Madison County focus group responses were predominately “no”, for example: “I’m not familiar with any of our local politicians promoting healthy lifestyles or healthy eating”. There was one exception: “I know that the mayor of Ridgeland, he’s a huge cyclist… I know the mayor does things to promote healthy lifestyles”. Rankin County participants pointed to the senior building as an example: “Yes, they promote, uh, activities because they built us a senior building”.

Overall, focus group participants’ perceptions of political leaders’ promotion of healthy eating and physical activity were mixed. They ranged from “no” among Madison County participants, to examples of support and/or participation for Hinds and Rankin Counties.

## 4. Discussion

During the last half of the 20th century and the early part of the 21st century, there were policy changes in the U.S. that were made to initiate environmental changes that later led to substantial suburban developments [17]. Many of these developments have changed the residential landscape in urban and suburban locations. Politicians and leaders in local cities and towns have the opportunity at their disposal to enact land-use policies, such as zoning regulations and building codes, to create community-wide environments that support physical activity [18]. In many areas around the country, policies have been devised to locate walking and physical activity facilities near neighborhoods, residential areas, shops, schools, and offices that can become available to community residents [19,20,21].

In 2002, the Centers for Disease Control and Prevention and the American Cancer Society convened the Fruit and Vegetable Environment, Policy, and Pricing Workshop. One of the goals of this workshop was to identify types of interventions, specific programs, and research needs related to environmental, policy, and pricing strategies to promote greater consumption of fruits and vegetables [22]. Other areas that have provided avenues for physical activities within communities have succeeded in doing so through the implementation of successful policy changes. The National Complete Streets Coalition believes that a comprehensive list of policies should be developed to enable local, state, and federal governments to be in a position to make streets safer for drivers, cyclists, and pedestrians, thereby providing opportunities for increased physical activity. Policies like these are needed in Mississippi communities that have existed without adequate facilities for healthy activities [23].

The trends noticed in physical activity in the U.S. have contributed to much of the morbidity and mortality recorded: In 2001, only about 18% of U.S. residents indicated that they walked as a form of transportation, and that number has been unchanged in the past decade [24]. Many community members in this study could not recall seeing or hearing of any local politicians promoting healthy lifestyles, such as physical activity or healthy eating. Even though it is generally accepted that consumption of fruits and vegetables is essential to promoting good health and an important factor in disease prevention [25], according to the majority of the community members, there has been limited interest and promotion by political leaders within most communities. Community members said that they have not heard any elected officials say anything about healthy eating. According to them, “If that was the case, we wouldn’t have so much obesity”. One person added: “One councilperson created or was instrumental in getting a walking trail in the neighborhood”. Over in Ridgeland, the mayor, who is an acclaimed cyclist, was influential in having a cycling trail added to Natchez Trace. He is one of the few who actually promotes healthy lifestyles. In Pearl, MS, there is a senior building that was established, and that may have been as a result of actions on the part of the political leaders.

The consensus is that political leaders need to promote healthy awareness that includes engagement in exercise and positive dietary practices. The participants believe that currently, the only thing that comes close to organized policy, is the programs implemented by the Jackson Heart Study Community Outreach Center (CORC). Through the Jackson Heart Study CORC, the communities have been exposed to and have become more involved in promoting healthy eating and physical activity.

Policy and environmental changes even on a national scale have significant effects on urban, suburban, and rural locations, such as the three counties that participated in this study (Hinds, Madison, and Rankin Counties). This research has underlying meaning and importance in these communities. There will be far-reaching implications if politicians choose to use policy and environmental action to initiate a plan of action that would facilitate and promote healthy lifestyles, such as physical activity or healthy eating, in the Mississippi communities.

Cardiovascular disease is the leading cause of death in Mississippi, accounting for over a third of all deaths in the state, and Mississippi's cardiovascular disease (CVD) mortality rate is the highest in the nation. As stated by the community members, inaccessibility to healthy foods and unavailability of adequate physical activity facilities accentuate the importance of engaging civic associations and civic coalitions to effect positive change in the community. Civic coalitions are needed to influence political action that can effectively address environmental challenges and can reduce the commercial activities that promote negative health outcomes. The implementation of effective policies and laws can achieve the desired changes, but such action must be propelled by concerted involvement by civic coalitions that can develop a public action plan designated to prevent or reduce the prevalence of cardiovascular disease. Politicians can increase their appeal to the community by teaming with alternative influencers, such as sports and entertainment figures, to stimulate the community further to accelerate their efforts to eliminate risk factors for chronic diseases. This expanded partnership would increase the effectiveness of the civic coalitions that can develop greater community support to tackle the policy and environmental changes that are needed to design a heart-healthy community. A potential partner for the Mississippi community is the Consortium for Southeastern Hypertension Control (COSEHC), a health care collaborative network that participates in the Transforming Clinical Practice Initiative. The Consortium for Southeastern Hypertension Control can provide technical assistance support to help equip clinicians in the Southeastern network region with the tools, information, and network support needed to improve quality of care and increase patients’ access to information. This partnership will be very significant in the Mississippi communities, especially since one of COSEHC’s primary goals is to improve cardiovascular disease health outcomes [26].

## 5. Conclusions

The results of this study demonstrated that Hinds, Madison, and Rankin Counties have not experienced much participation from political leaders in health prevention/intervention efforts. Even though there was evidence of isolated, individual administrative involvement of one or two local politicians in health promotion issues, this proactive approach did not occur on a large scale. Mississippi’s current health crises put a lot of stress on the communities and the health care system. Unless adequate interventions are made to reverse the situation, it is likely that the long-term overall effect will continue to be disastrous for future generations.

Since it is well established that policy decisions have to be made to influence and facilitate environmental changes to address most health disparities, it is important that civic groups take the steps necessary to form the coalitions that can impact such change. These coalitions should include stakeholders, such as community residents, families, schools, and other organizations. These community groups must convene for the purpose of gathering information, engaging the general public, and engaging the policy-makers, such as the mayor, city council, legislative members and state and national representatives, for the purpose of developing and implementing policies dedicated to addressing community concerns regarding environmental and health concerns.

Geographic variation and available opportunities [22] must be considered when communities come together with lawmakers to plan for developing policy changes to impact health outcomes in Mississippi. Policy application can influence grocery store pricing to ensure reduced prices, increased availability of healthy commodities, adequate information regarding nutritional values of foods, and accessibility of the local community to variety and choice. The absence of these options can limit the community’s control of their own health destiny. The promotion of civic coalitions can help to promote sustainability in transitioning from negative to positive behaviors, while helping community members to reduce risk behaviors. The availability of healthy foods and products in neighborhood stores has long been associated with healthy behaviors and positive health outcomes [23]. This can make a difference in the Mississippi communities where supermarkets are not accessible and health disparities abound [27].

## Figures and Tables

**Table 1:** Focus Group Questions.

Focus Group Questions
When you think about your political leaders that you have in your community, do any of them promote healthy eating and physical activity?

**Table 2:** Community members’ perception of political leaders’ involvement in healthy living.

Themes/Subthemes	Counties
**Built Environment**	
Walking Trail developed based on a council person’s support and recommendation.	H
Cycling trail was added to Natchez Trace.	M
They built a senior building to promote healthy living.	R
There is limited interest and promotion by political leaders within most the communities.	H, M, R
**Promotion of Healthy Living**	
Some interest by political leaders in promoting healthy eating and physical activity.	H
The Jackson and Ridgeland mayor have participated in several walks throughout the city.	H, M
Majority reported that political leaders in their community do not promote healthy eating and physical activity.	H, M, R

H: Hinds County; M: Madison County; R: Rankin County.
